# Multi-skill resource-constrained multi-modal project scheduling problem based on hybrid quantum algorithm

**DOI:** 10.1038/s41598-023-45970-y

**Published:** 2023-10-28

**Authors:** Jun Long Peng, Xiao Liu, Chao Peng, Yu Shao

**Affiliations:** 1 Changsha University of Science & Technology, Changsha, People’s Republic of China; 2 China State Construction Hailong Technology, Shenzhen City, China

**Keywords:** Civil engineering, Computational science

## Abstract

Numerous studies on project scheduling only consider a single factor, which fails to reflect the actual environment of project operations. In light of this issue, the article synthesizes multiple perspectives and proposes a multi-skill resource-based multi-modal project scheduling problem (MRCMPSP). This problem is described, modeled, and solved using the resource capability matrix and other constraints to minimize the project duration. To effectively solve MRCMPSP and enrich scheduling algorithms, the paper selects the hybrid quantum algorithm (HQPSO) based on the quantum particle swarm algorithm (QPSO). The HQPSO introduces various improvements such as the JAYA optimization search to improve the algorithm's performance. In order to verify the generality, superiority, and effectiveness of the algorithm, independent operation comparison experiments and practical application experiments of the algorithm are designed based on different case sizes and resource quantities. The experimental results demonstrate that the proposed algorithm has superior convergence performance and solution accuracy and can provide an effective scheduling solution for real cases. Additionally, the article provides targeted management suggestions based on the research findings. Overall, this study contributes a novel mathematical model, solution algorithm, optimization strategies, and managerial insights, advancing the field of project management research.

## Introduction

In today's fast-paced world of science and technology, companies are engaged in fierce competition, and a company's competitiveness is often directly linked to the efficiency and quality of its project completion. A recent survey indicates that over 70% of construction projects are delayed, with 75% of them incurring actual costs that surpass 50% of the budgeted costs due to scheduling delays^1^. Therefore, it is crucial for companies to manage and schedule their projects effectively. A well-planned project schedule can save valuable resources, such as manpower, and help complete projects efficiently and smoothly. In this competitive environment, researchers have begun to focus on the resource-constrained project scheduling problem (RCPSP) as a highly representative class of project scheduling problems. RCPSP is also an abstract representation of many practical project scheduling problems and has been proven to be an NP-hard problem^2^, which focuses on how to effectively deploy each process and resource supply to achieve the optimization goal of the shortest project completion time based on satisfying the logical constraints between each process and resource constraints^3^ . However, as the research on the resource-constrained project scheduling problem continues to advance, the traditional resource-constrained project scheduling problem can no longer meet the research needs of today's researchers, who are gradually working to improve the theory of the scheduling problem to make the problem more suitable for real project environments and very challenging, so researchers are combining the theoretical problem with the real environment^4^. For instance, in response to the need for more practical research, traditional resource-constrained project scheduling problems have been expanded to include non-preemptive multi-skill resources such as human resources, resulting in the Multi-Skill Resource Constrained Project Scheduling Problem (MCRCPSP). The MCRCPSP is an extension of the RCPSP, and it not only involves planning resource and process deployment but also requires optimal allocation of resource skills. Moreover, in actual project operations, researchers have discovered that there are multiple modes of process execution, and the duration of project activities varies depending on the process execution mode. This led to the emergence of the Multi-Modal Resource Constrained Project Scheduling Problem (MRCPSP), which investigates how to select the project process execution mode and optimize resource deployment accordingly^5^. In addition to theoretical extensions, designing a superior algorithm for solving RCPSP and its variants is also an urgent problem for project scheduling research today^6,7^. At present, both domestic and foreign solution methods mainly consist of exact optimization algorithms and heuristic optimization algorithms. However, exact optimization algorithms are often time-consuming and inefficient, while heuristic algorithms have become the focus of researchers due to their high solution efficiency^8–11^. Among them, the quantum particle swarm algorithm (QPSO), as an extremely excellent heuristic algorithm, is based on the particle swarm algorithm (PSO) and invokes the principle of quantum mechanics, which can effectively improve the global search performance as well as the operation speed with few control parameters of the algorithm, but in the process of particle evolution, there is still the problem of premature convergence, leading to too slow convergence of the algorithm in seeking the global optimal solution.

Therefore, to increase the applicability of the project scheduling problem to real-world environments, this paper integrates the characteristics of multi-modal projects and multi-skilled resources. The study proposes a more complex and valuable research problem, namely the multi-skill resource-constrained multi-modal project scheduling problem (MRCMPSP), based on the multi-skill resource-constrained project scheduling problem (MRCPSP). Furthermore, the article introduces a hybrid quantum algorithm that is built on QPSO and incorporates various improvements with the goal of effectively solving these problems.

Based on the above analysis, the main contributions of this article are as follows:

1. In practical applications, the article uses an improved quantum particle swarm algorithm to solve a multi-modal project scheduling problem involving multi-skilled resource constraints. Based on the experimental results, corresponding management recommendations are also presented. By bridging the gap between practical implementation and theoretical research, this study provides a more efficient and optimized scheduling solution for production, leading to increased productivity and economic efficiency.
2. In terms of theoretical research, given the limited coverage of existing literature on project scheduling problems with integrated resource constraints, this paper designs a novel non-linear mathematical model for multi-modal project scheduling problems with multi-skilled resource constraints. The aim is to enrich project scheduling theory. Furthermore, the application of the improved quantum particle swarm algorithm to the scheduling problem is extended by combining it with the problem. This in-depth exploration of integrated resource scheduling methods provides valuable references and inspiration for further research and practice in related academic fields.
3. In terms of algorithm optimization, this paper introduces a variety of improvements, such as JAYA optimization search, which significantly improves the performance of the algorithm and provides guidance to other researchers seeking to improve the performance of the algorithm.

## Literature review

### Project scheduling technology development

Modern project scheduling techniques originated in the United States, including the Critical Path Method (CPM) and Program Review Method (PERT), which were used in famous projects such as the Manhattan Project, Polaris Missile Program, and Apollo Moon Program^12^. However, as the earliest representative of scheduling technology, CPM/PERT does not consider resource constraints, resulting in a resource scheduling plan that is disconnected from the actual project plan. Consequently, project plans executed according to CPM/PERT are frequently delayed due to resource constraints, resulting in the failure to complete the project on time. In response, the Resource Constrained Project Scheduling Problem (RCPSP), a class of project scheduling techniques that consider resource constraints, was introduced Kelley to produce a schedule that optimizes management objectives while satisfying activity tightness and resource constraints^13^. Since then, many scholars have launched the solution for RCPSP, for example, Amir developed a convolutional neural network method to solve RCPSP and investigated the performance of the convolutional neural network (CNN) method using standard benchmark problems in PSPLIB and compared it with the MLFNN method and standard meta-heuristics^14^; Feng et al. proposed an extended genetic algorithm for solving the RCPSP, and experiments showed that the extended genetic algorithm can solve the RCPSP faster and more accurately than the traditional genetic algorithm^15^. For example, Hua et al. proposed an improved genetic algorithm based on time window decomposition in order to solve the RCPSP problem more efficiently, which employs three derived methods to increase population diversity^16^. Bettemir et al. proposed a hybrid algorithm based on a genetic algorithm and simulated annealing for RCPSP to achieve an efficient solution of RCPSP^9^. Liu et al. chose the genetic algorithm to solve the RCPSP problem and proved that it is a highly competitive algorithm for solving the RCPSP^10^.

### A review of the literature related to the resource-constrained project scheduling problem and its variants

In practical project scheduling problems, the existence of differences in the execution mode of process activities makes resource requirements and process durations diverse, which greatly increases the practicality and difficulty of solving scheduling optimization problems^17^. Therefore, researchers have started to consider the impact of process multimodality on the solution of project scheduling problems in a comprehensive manner. For example, David provides an overview of compact continuous-time formulations of the multi-modal resource-constrained project scheduling problem from previous studies and considers an equivalent reformulation with sparse constraint matrices on the basis of the previous research formulations, which in turn reconstructs the multi-modal resource-constrained project scheduling mode^18^. Zoraghi performed objective optimization for a multi-modal project scheduling problem with a model optimization objective of minimizing the project cost and selected a hybrid heuristic optimization algorithm to solve the corresponding problem^19^. Cheng et al. used a branch-and-bound optimization algorithm to solve the scheduling model based on the multi-modal project scheduling problem, taking into account the lead time and completion time of the active processes^20^. Oztemel & Selam solved MRCPSP with the help of a swarm optimization algorithm using an injection mold manufacturing project as a case study and applied the algorithm to similar project scheduling problems of different sizes^21^. The multi-modal resource-constrained multi-project scheduling problem was considered by Asta, and selected a two-stage optimization algorithm based on Monte Carlo Tree Search (MCTS) to solve the problem^22^. Chen et al. proposed a hybrid genetic algorithm (HGA) and heuristic-based approach to solve the resource-constrained multimodal scheduling problem, and experimentally demonstrated that the HGA algorithm using a parallel approach and a minimum relaxation priority rule outperforms simple genetic algorithms in the existing literature^23^. In the project scheduling problem, in addition to considering the impact of factors such as the ability to update resources as suggested by Slowinski^24^. How to fully consider multi-skill resources in the project scheduling problem is certainly of great research interest. For example, Daniels & Mazzola found that the rational allocation of multi-skill resources can greatly improve the production performance of the flow shop, and proposed a branch boundary optimization algorithm and heuristic optimization algorithm to solve the strong NP-hard problem of the flow shop scheduling with limited Multi-Capability Resources^25^. Li et al. proposed a multi-objective JAYA algorithm for the multi-skilled resource-constrained project scheduling problem with the objective of minimizing production and total costs^26^. Behrad et al. proposed an integer linear programming model for scheduling multi-skilled resource-constrained projects and developed genetic (GA) and simulated annealing (SA) meta-heuristic algorithms to solve the model^27^. Peng et al. proposed a preemptive multi-skill resource-constrained project scheduling problem and a task-oriented scheduling model for marine power equipment maintenance with shipbuilding industry as the research object, and designed an improved moth-flame optimization algorithm, and finally proved the effectiveness of the proposed algorithm by comparing the experimental results^28^.

## Research gap analysis

The aforementioned studies collectively offer valuable insights and recommendations for the implementation of multi-modal project scheduling, particularly in the context of multi-skill resource constraints. In contrast to conventional project scheduling techniques, our approach in this paper takes into account the critical factor of resource constraints.

Moreover, a thorough review of existing literature reveals a notable scarcity of comprehensive investigations pertaining to multi-skill resources and multi-modal projects within the traditional Resource-Constrained Project Scheduling Problem (RCPSP) framework.

Regarding the choice of solution method, the article adopts the quantum particle swarm algorithm. As a heuristic optimization technique, the quantum particle swarm algorithm not only has powerful search capability, but also has fewer control parameters. As shown by the above literature analysis, the application of quantum particle swarm algorithm to the integrated scheduling problem is still not fully explored in the current research.

Additionally, to enhance the performance of our algorithm and the quality of its solutions, our article introduces optimization techniques such as the JAYA optimization search mechanism, aimed at efficiently optimizing the scheduling problem.

## Method

**Figure 1:**
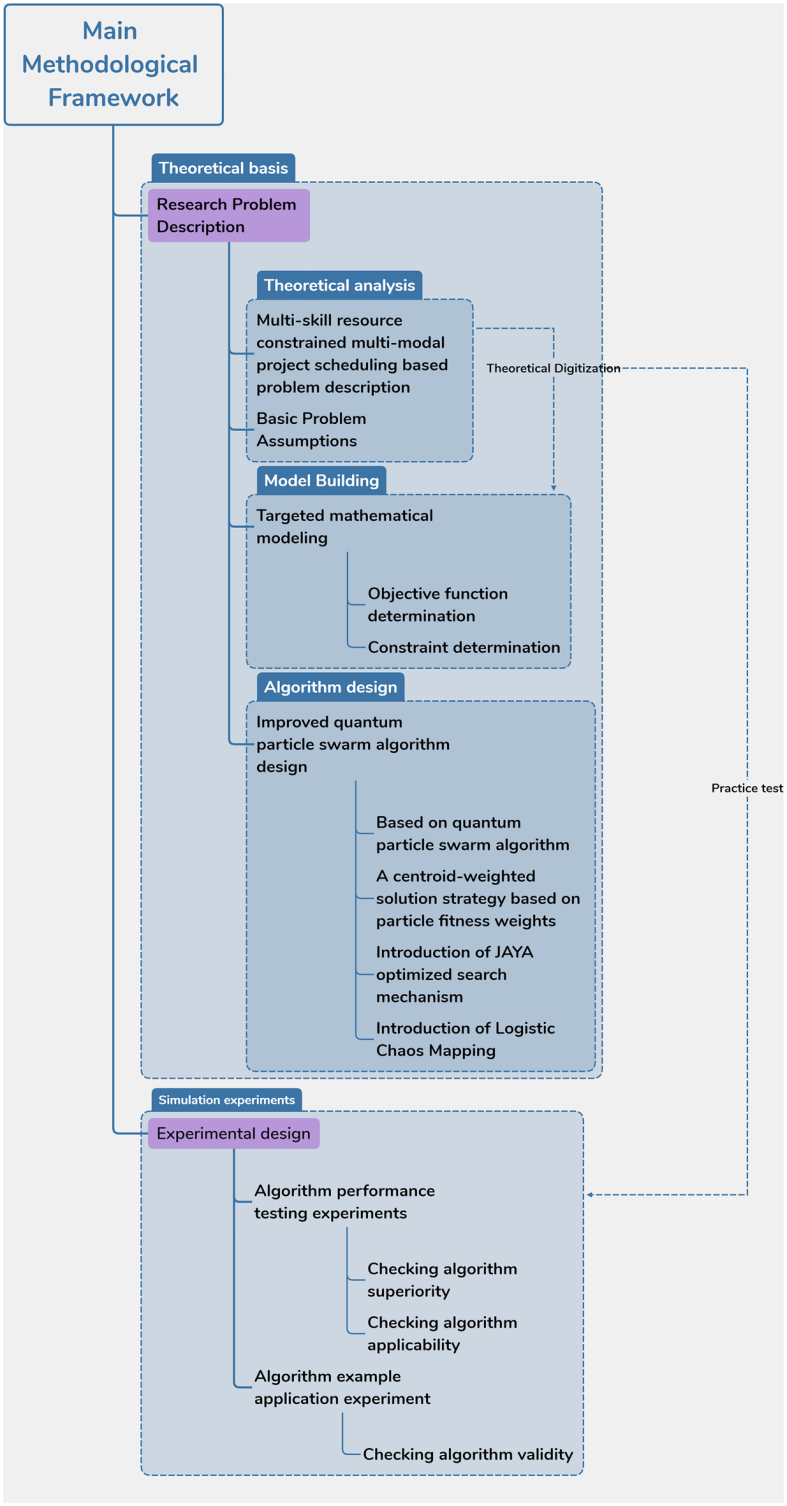
Main methods and technical framework.

This section details the research methodology of this paper, which is divided into two main parts: theoretical basis, simulation and simulation, and the main methodological framework is detailed in Fig. 1 below.

### Multi-mode project scheduling description based on multi-capability resources

The scheduling problem addressed in this paper is represented by a directed acyclic graph (AON) denoted as G = (V, E). The AON graph effectively captures the logical relationships between project activity processes, specifically the immediately preceding and following relationships. In this representation, G signifies the set of nodes within the graph, where each node corresponds to a sub-process involved in project execution. V = [0, 1, 2, …, n1, n2, …, n] represents the collection of non-preemptive active processes. Furthermore, E represents the set of directed arcs that depict the dependency relationships between each process.

**Figure 2:**
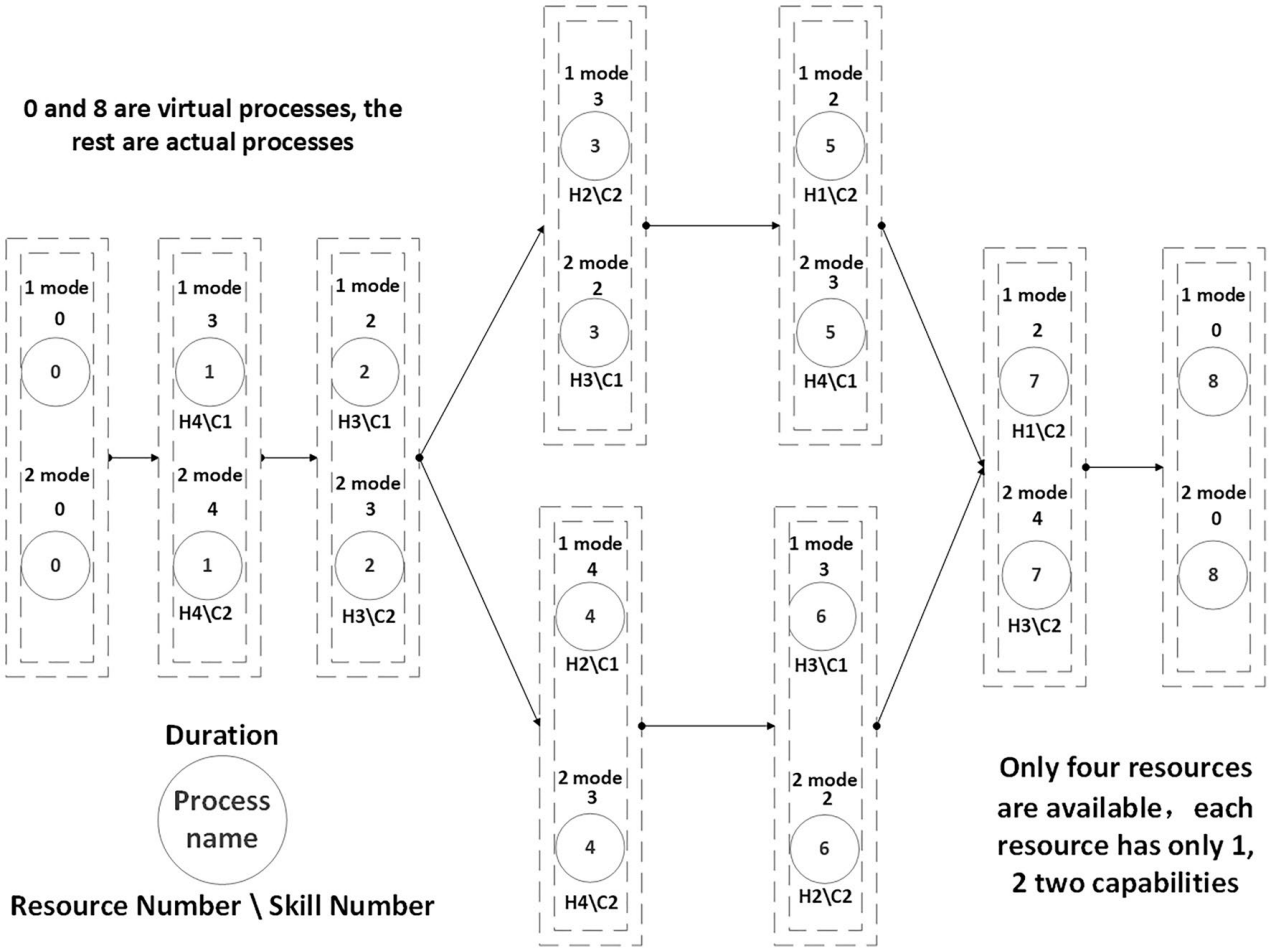
An example of multi-mode project scheduling based on multi-skill resource constraints.

Activity processes 0 and n are virtual processes symbolizing the project's start and end, respectively. These two processes do not consume any resources or require execution time. The project activity processes necessitate a set of skills denoted as C = {1, …, c}. These capabilities are fulfilled and executed by a set of resources required for the project activity, denoted as H = {1, …, h} (where h ∈ H). For clarity, Fig. 2 showcases an illustrative example of multi-modal project scheduling based on multi-skilled resource constraints. Figure 2 shows an example of multi-modal project scheduling based on multi-skilled resource constrained.

### Basic assumptions

To simplify the practical problems, it is necessary to combine the relevant theoretical literature and the corresponding requirements of modern enterprise projects before designing the multi-mode project scheduling model based on multi-capability resources. Otherwise, the problem under consideration is overly complex; on the one hand, it is inconvenient to calculate and difficult to solve; on the other hand, the results cannot be tailored to the needs of various enterprises on a large scale^29^. This paper will make the following basic assumptions on the resource-constrained project scheduling model after summarizing and summarizing previous kinds of literature^29,30^:

1. An active process cannot be executed until all the immediately preceding processes of the active process have been completed;
2. The non-possession of resources means that only after the current activity process has been executed can the resources execute another activity process;
3. The project activity process must meet its skill requirements during its execution period;
4. Every resource is renewable in the process of project execution;
5. In the process of project execution, only one execution mode can be selected for each process.

### Establishment of continuous mathematical model

In order to facilitate the establishment of the follow-up model, this paper summarizes the mathematical symbols and symbolic descriptions used in the model, and explains each symbol separately, as shown in the following Table 1:

**Table 1:** Project operation execution mode-duration table.

Symbols	Meaning
V	Process collection
C	Ability collection
H	Collection of resources
n, n1, n2	Operation index
c	Ability index
h	Resource index
s	Pattern index
Sn	Number of execution modes of n operation, n ∈ V
$\mathrm{pcm}(\mathrm{h},\mathrm{c})=\{\mathrm{0,1}\}$	resource capability matrix, indicating whether resource h has the capability c, h ∈ H, c ∈ C
$\mathrm{P}(\mathrm{n}1,\mathrm{n}2)=\{\mathrm{0,1}\}$	Process logic relation matrix, judging whether n1 is the immediately preceding process of n2, n1, n2 ∈ V
$\mathrm{T}\left(\mathrm{n},\mathrm{s}\right)$	Mode-time matrix, which indicates the time of operation N in execution mode S, n ∈ V, S ∈ {1,2,3 … Sn}
$D(c,n,s)$	Skill-mode matrix, which indicates the demand quantity of operation c for skill c in mode S, n ∈ V, s ∈ {1,2,3 … Sn}, c ∈ C

(1) The decision variables and dependent variables in the mathematical model are shown below:

1 $$X\left( {n,\;s} \right) = \left\{ {\begin{array}{*{20}l} {1,} \hfill & {If \;the \;process\; n \;selects\; the \;mode\; s \;to\; execute \;the\; process} \hfill \\ {0,} \hfill & {On\; the \;contrary} \hfill \\ \end{array} } \right.$$

2 $${\text{Y}}\left( {{\text{h}},\;{\text{n}},\;{\text{c}}} \right) = \left\{ {\begin{array}{*{20}l} {1,} \hfill & {If \;process\; n\; uses\; resource\; h\; to \;perform\; its\; capability \;c} \hfill \\ {0,} \hfill & {On \;the \;contrary} \hfill \\ \end{array} } \right.$$

3 $$T\left( {en} \right):\; Start \, \;time \, \;of \, \;operation \, \;n,$$

4 $$T\left( {un} \right):\;End\; \, time\; \, of\; \, operation\; \, n,$$

(2) Target function setting

5 $$Min\;F,\; F = Max\{ T\left( {un} \right)|n \in V\}$$

(3) Constraint setting

6 $$\forall n \in V,\;Sn \ge 1,\;Sn \in Z$$

7 $$\forall n \in V,\;\forall s \in \left\{ {1,\;2,\;...,\;Sn} \right\},\;\forall c \in C,\;D\left( {c,\;n,\;s} \right) \ge 0,\;D\left( {c,\;n,\;s} \right) \in Z$$

8 $$\forall n \in V,\;T\left( {en} \right) \ge 0,\;T\left( {un} \right) \ge 0$$

9 $$\mathop \sum \limits_{c \in C} pcm\left( {h,\;c} \right) \ge 1,\;\forall h \in H,\;\forall c \in C$$

10 $$\forall n1 \in V,\;\forall n2 \in V,\;P\left( {n1,\;n2} \right) = 1$$

11 $$\forall n1 \in V,\;\forall n2 \in V,\;T\left( {en2} \right) - T\left( {un1} \right) \ge 0$$

12 $$T\left( {un} \right) = T\left( {en} \right) + \mathop \sum \limits_{{s \in 1,2,..S_{n} }} T\left( {n,\;s} \right)*X\left( {n,\;s} \right),\;\forall n \in V$$

13 $$\mathop \sum \limits_{s \in 1,2,..Sn} X\left( {n,\;s} \right) = 1,\;\forall n \in V$$

14 $$\forall c \in C,\;Y\left( {h,\;n,\;c} \right) \le pcm\left( {h,\;c} \right),\;\forall n \in V,\;\forall h \in H$$

15 $$\mathop \sum \limits_{c \in C} Y\left( {h,\;n,\;c} \right) \le 1,\;\forall n \in V,\;\forall h \in H$$

16 $$\mathop \sum \limits_{h \in H} Y\left( {h,\;n,\;c} \right) = \mathop \sum \limits_{s \in 1,2,..Sn} D\left( {c,\;n,\;s} \right)*X\left( {n,\;s} \right),\;\forall n \in V,\;\forall c \in C$$

17 $$\mathop \sum \limits_{c \in C} Y\left( {h,\;n1,\;c} \right) = 1,\mathop \sum \limits_{c \in C} Y\left( {h,\;n2,\;c} \right) = 1$$

18 $$\max \left( {T\left( {en1} \right),\;T\left( {en2} \right)} \right) - \min \left( {T\left( {un1} \right),\;T\left( {un2} \right)} \right) \ge 0$$

19 $$\forall h \in H,\;\forall n1 \in V,\;\forall n2 \in V,\;n1 \ne n2$$

Equations (6), (7), and (8) represent the value ranges of variables and related data in the model; Formula (9) indicates that each resource has at least one capability in the project activities; Eqs. (10) and (11) indicate the time sequence constraint, that is, the active process can only be started after all the immediately preceding operations are completed; Formula (12) indicates the calculation of the execution end time of the project activity process; Formula (13) indicates the mode constraint, that is, only one mode can be selected for the project activity process; Eq. (14) indicates that only resources with corresponding capabilities can be used in the process of process execution; Formula (15) indicates that each resource can only use one capability to perform the activity operation; Formula (16) shows that it can meet the requirements of each activity process for skill; Eqs. (17), (18), and (19) indicate that resources can't be preempted, that is, the resources can only execute another activity process after the execution of the activation process.

### Design of hybrid quantum algorithms

Since the performance of the exact solution optimization algorithm is susceptible to the influence of instance size, however, in practical situations, the actual case sizes of project scheduling problems are large^1,4,6,7,9,10,31^. In addition, because the multi-modal project scheduling problem based on multi-skill resources studied in this paper integrates multi-skill resources and the multi-modality of project activity processes, the decision factors and constraints of the traditional RCPSP are increased, which leads to a more complex problem model and the distribution of feasible solutions is full of diversity. Therefore, it is of great research significance to seek an effective heuristic optimization algorithm with efficient problem-solving ability. In this section, a hybrid quantum algorithm is designed for the Multi-Capability Resources-constrained multimodal project scheduling problem.

#### Principle of Standard Quantum Particle Swarm Optimization

QPSO algorithm is a swarm intelligent optimization algorithm with global search capability. It is based on the PSO algorithm and refers to the particle motion law in quantum mechanics principle, which effectively improves the global search performance and operation speed with fewer control parameters^4,32–35^. Therefore, to solve this problem more effectively, this paper designs an improved hybrid quantum algorithm based on quantum particle swarm optimization.

Compared with the PSO algorithm, in the QPSO algorithm, particles only have position information, and the position update is determined by the following three equations:

Let the population size of the particle be N, the iteration step of the algorithm is the t step, the particle moves in D-dimensional space, the potential well of the particle in the d dimension is $p(id)(\mathrm{t})$, the update equation of the particle x(t) can be described as

20 $$x\left( {id} \right)\left( {t + 1} \right) = p\left( {id} \right)\left( t \right) \pm \gamma \left| {B\left( d \right)\left( t \right) - x\left( {id} \right)\left( t \right)} \right|*\ln \left( {1/u\left( {id} \right)\left( t \right)} \right)$$

21 $$p\left( {id} \right)\left( t \right) = \partial \left( t \right)*p\left( {id} \right)\left( t \right) + \left( {1 - \partial \left( t \right)} \right)*G\left( d \right)\left( t \right)$$

22 $$B\left( t \right) = \left( {B\left( 1 \right)\left( t \right),\;B\left( 2 \right)\left( t \right),\;B\left( 3 \right)\left( t \right),\;B\left( 4 \right)\left( t \right),\;...,\;B\left( D \right)\left( t \right)} \right)$$

23 $$B\left( t \right) = \left( {1/N} \right)*\mathop \sum \limits_{i = 1}^{N} P\left( i \right)\left( t \right)$$

where P(i)(t) is the current optimal position of the i-th particle, and G(t) and B(t) are the global optimal position and the average optimal position of the particle population; ∂ (t) and u(id)(t) are uniformly distributed random numbers in the interval of [0,1].

#### Design of a hybrid quantum particle swarm optimization algorithm

Although the traditional QPSO algorithm no longer uses the PSO algorithm's speed and position update strategy, and it has better global convergence than PSO with less parameter consideration, there are still some issues when it is applied to Multi-Capability Resources-constrained multi-mode project scheduling, such as the decline of population diversity in the later stage, which reduces the diversity of non-dominated solutions^32^. Furthermore, it is easy to fall into local optimal under the guidance of historical global optimal particles, leading to premature convergence. To compensate for the aforementioned flaws, we will make the following algorithm improvements in this section:

1. The particle fitness weight is introduced to calculate the center point, that is, the optimal position of the average particle;
2. The search mechanism of the JAYA algorithm is introduced to enhance the single particle search ability and global search performance;
3. logistic chaotic map is introduced to conduct a chaotic search on individuals with 15% population fitness, to generate new solutions, thus improving the diversity of non-dominated solutions, the global search performance, and the convergence accuracy of the algorithm;

(1) A centroid-weighted solution strategy based on particle fitness weights.

The center point introduced by the traditional QPSO algorithm, namely the average optimal position, is calculated by the following formula:

24 $$B\left( t \right) = \left( {1/N} \right)*\mathop \sum \limits_{i = 1}^{N} P\left( i \right)\left( t \right)$$

However, this formula adopts the idea of mean value to solve the center point, but because of the difference in fitness of each particle, there are some limitations in solving the average optimal position by this idea, that is, it can't be closer to the optimal direction. In this study, we introduce the individual fitness weight to solve the center point, which makes the center point more inclined in the optimal direction. The specific formula is as follows:

25 $$B\left( t \right) = \left( {1/N} \right)*\mathop \sum \limits_{i = 1}^{N} \theta \left( i \right)*P\left( i \right)\left( t \right)$$

26 $$\theta \left( i \right) = Q\left( i \right)\left( t \right)/\mathop \sum \limits_{i = 1}^{N} Q\left( i \right)\left( t \right)$$

where θ_i_ represents the weight normalized by each particle's fitness, which reflects the influence of each particle's fitness on the centroid. When the weight of a particle adaptation is larger, the influence of the particle adaptation on the centroid is also larger; Q(i)(t) is the fitness of the i-th particle; ${\sum }_{\mathrm{i}=1}^{\mathrm{N}}\mathrm{Q}(\mathrm{i})(\mathrm{t})$ represents the sum of all particle finesses within the quantum particle population.

(2) JAYA Optimized Search.

This paper introduces JAYA search as a way to enhance the local search performance and solution accuracy of the algorithm. The iterative equation of JAYA optimization search is as follows^36–38^:

27 $$\begin{aligned} X\left( {i,\;k,\;\left( {t + 1} \right)} \right) = & X\left( {i,\;k,\;t} \right) + r\left( {best,\;i,\;k,\;t} \right)*(X\left( {best,\;k,\;t} \right) - \left| {X\left( {i,\;k,\;t} \right)} \right|) \\ & - r\left( {worst,\;i,\;k} \right)*(X\left( {worst,\;i,\;k,\;t} \right) - \left| {X\left( {i,\;k,\;t} \right)} \right|) \\ \end{aligned}$$

**Figure 3:**
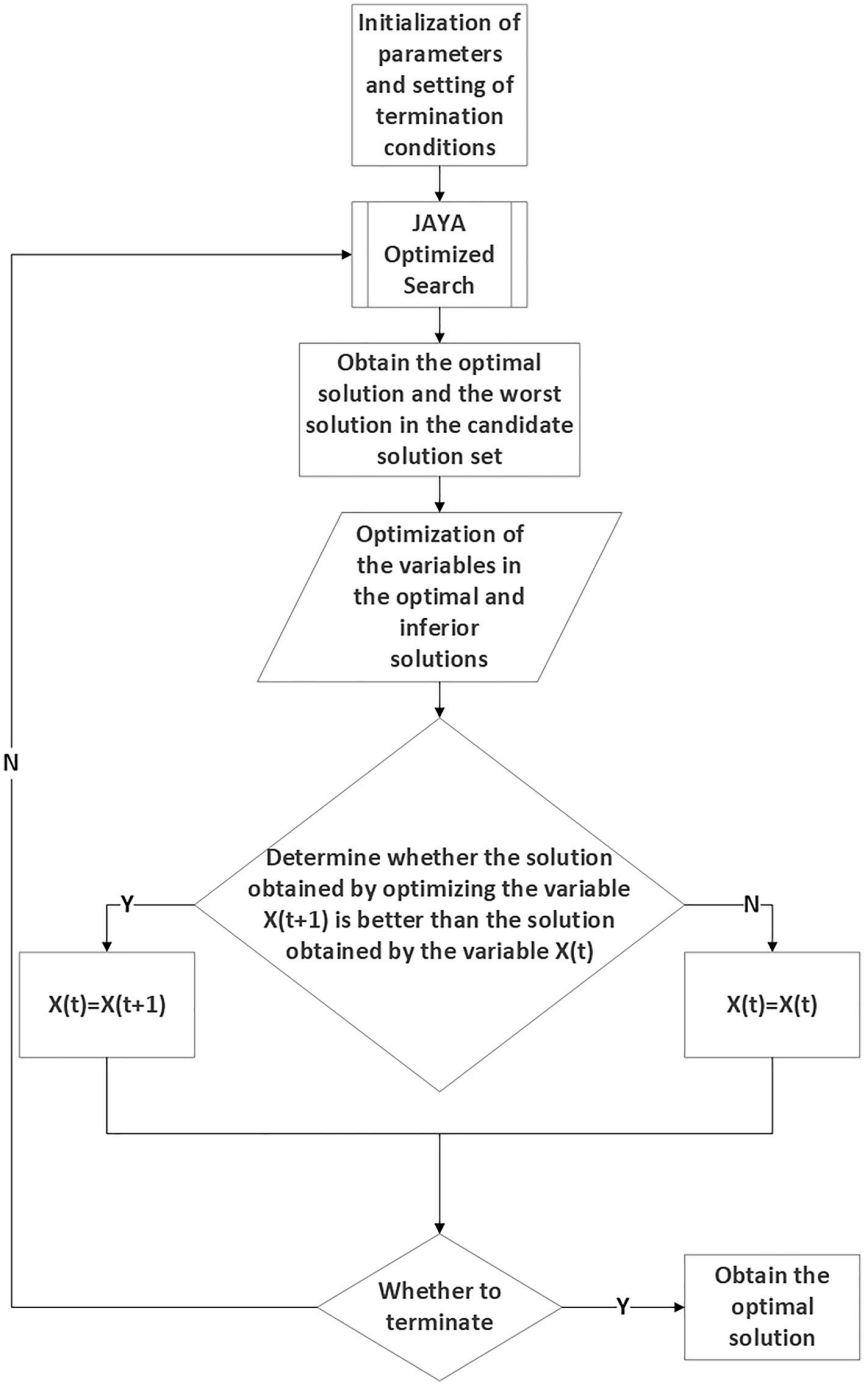
JAYA Optimized Search Process.

"i" represents the i-th quantum particle in the population, where $i \in [\mathrm{1,2}, ... n+1]$."k" represents the k-th dimensional variable of the quantum particle, where $\mathrm{k }\in [1, 2, ...\mathrm{ d}]$."t" represents the current iteration times of the algorithm."X(t) " and "$\mathrm{X}(\mathrm{t}+1)$" respectively represent the updated values of the i-th quantum particle in the t-th iteration before and after iterative calculation by the JAYA search optimization formula on the k-th dimension."$\mathrm{r}(\mathrm{best})$" and "$\mathrm{r}(\mathrm{worst})$" are random numbers within the interval [0, 1]. Adjusting these parameters allows for fine-tuning the ability of quantum particles to approach the optimal solution." $\mathrm{X}(\mathrm{best},\mathrm{k},\mathrm{t})$" and "$\mathrm{X}(\mathrm{worst},\mathrm{i},\mathrm{k},\mathrm{t})$" represent the values of the best and worst quantum particles in the n-th iteration in the k-th dimension." $\mathrm{r}(\mathrm{best},\mathrm{i},\mathrm{k},\mathrm{t})*(\mathrm{X}\left(\mathrm{best},\mathrm{k},\mathrm{t}\right)-\left|\mathrm{X}\left(\mathrm{i},\mathrm{k},\mathrm{t}\right)\right|)$" Indicates the evolution of the current individual towards the contemporary optimal quantum particle." $\mathrm{r}(\mathrm{worst},\mathrm{i},\mathrm{k})*(\mathrm{X}(\mathrm{worst},\mathrm{i},\mathrm{k},\mathrm{t})-|\mathrm{X}(\mathrm{i},\mathrm{k},\mathrm{t})|)$" Implies keeping the current quantum particle away from the worst contemporary individual. If the fitness of the newly generated quantum particle is superior to that of the original quantum particle, the original quantum particle is replaced by the new one. Otherwise, the JAYA optimization iteration continues for the next quantum particle. Once all quantum particles have been traversed, the next round of optimization search iteration is carried out. For a visual representation of the specific process, please refer to Fig. 3.

(3) logistic chaotic mapping.

The initialization of the particle population plays a crucial role in determining the convergence speed and solution quality of the algorithm^39^. Typically, particle swarm algorithms and their variants rely on uniformly distributed random initialization of the population^40^. However, traditional QPSO optimization algorithms heavily rely on the historical global best particles obtained through external selection to update the population, which may lead to a lack of diversity in the population during the later stages of evolution^41^.

To address this issue, researchers such as Lu et al. demonstrated in 2014 that incorporating chaotic mapping can effectively prevent the algorithm from getting trapped in local optima^41^. Chaotic mappings, such as logistic mappings or tent mappings, are commonly used in this context^42^. Logistic mappings, in particular, are often utilized in designing chaotic flow cryptosystems due to their proven security properties.

To enhance the diversity of non-dominated solutions and improve the accuracy of the algorithm, this study introduces the use of logistic chaos mapping. Specifically, the logistic equation is employed to generate new solutions by applying chaos mapping to individuals in the bottom 15% of the population in terms of fitness. The core concept behind chaos mapping is to generate chaotic sequences iteratively, and logistic chaos mapping accomplishes this by utilizing logistic equations:

28 $$y\left( {t + 1} \right) = \mu *y\left( t \right)*\left[ {1 - y\left( t \right)} \right]$$

**Figure 4:**
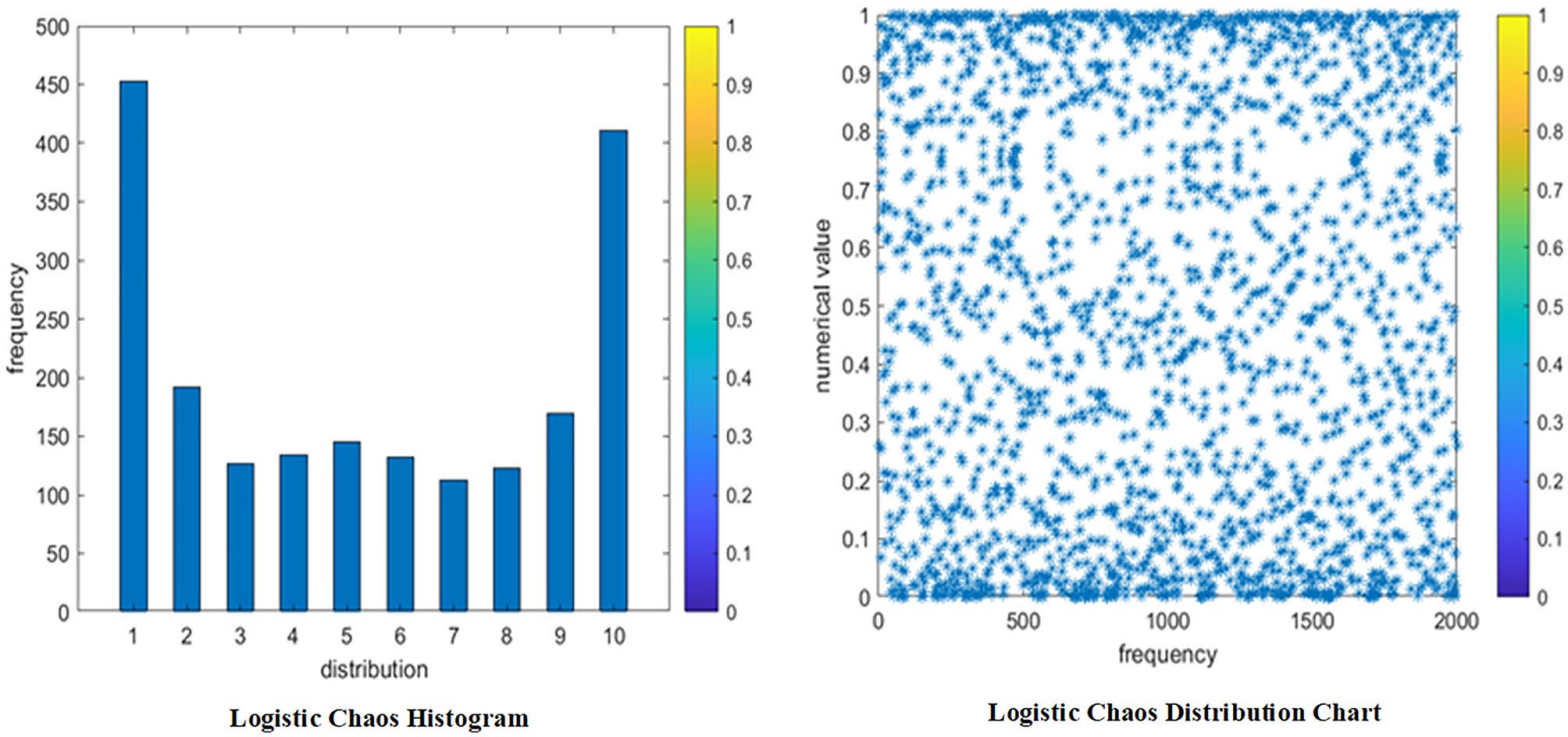
Logistic Chaos Histogram.

It is proved experimentally that when the bifurcation parameter μ = [3.57,4], the Logistic mapping is in a completely chaotic state, and the trajectories of the equations in this interval show completely chaotic characteristics^42^. Figure 4 shows the histogram and distribution of the logistic chaos mapping for this study, respectively. Moreover, it is found by this experiment that better results can be obtained when μ is taken as 4. Therefore, μ = 4 in the new hybrid quantum particle swarm algorithm proposed in this paper.

#### Algorithm steps

Step1: Determine the values of population size, number of iterations, and other parameters;

Step2: Read the project activity process base data (including logical relationships, resource skill matrix, model duration matrix, etc.);

Step3: Initial population generation;

Step4: Calculate the particle fitness value, update the particle historical optimal point and population optimal point;

Step5: determine whether the conditions of the termination algorithm are met and if so, output the result; otherwise, go to the next step;

Step6: quantum particle swarm centroids calculated based on the particle fitness weights and complete the update of the average particle optimal position;

Step7: updating the population based on the quantum particle population position update formula;

Step8: Perform a search for new individuals using the JAYA algorithm search mechanism and compare them with the new individuals generated by the quantum particle swarm to retain the better ones, thus completing the iterative update;

Step9: Chaos mapping is applied to the individuals after 15% adaptation using logistic mapping, to generate new individuals and finally return to step5.

**Figure 5:**
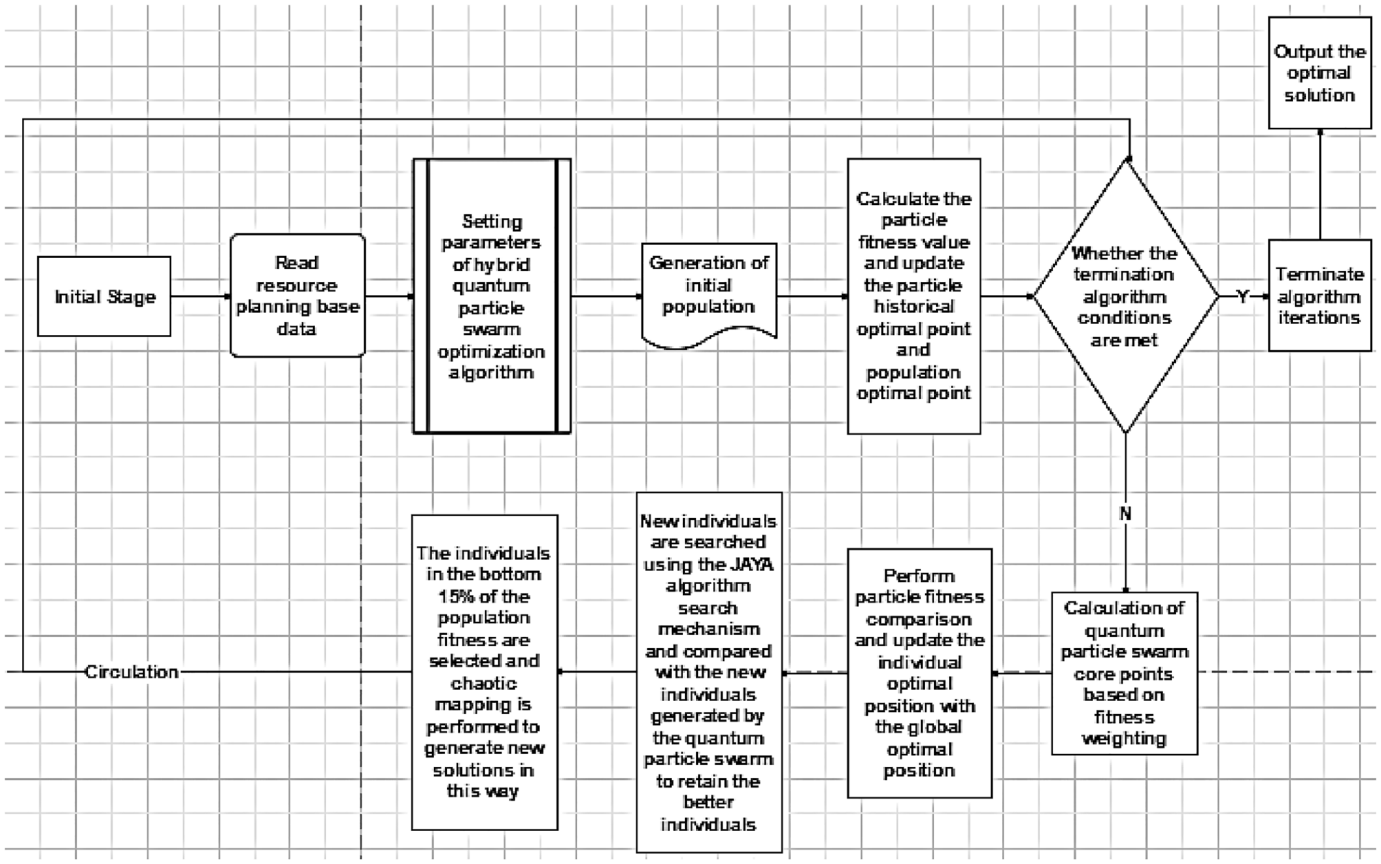
JAYA-Chaos Mapping-Quantum Particle Swarm Hybrid Optimization Algorithm Flowchart.

The specific flow of the hybrid quantum particle swarm algorithm is shown in Fig. 5.

## Algorithm performance testing

### Test case adjustment

PSPLIB, an international standard library of arithmetic cases, contains a large number of test cases for project scheduling algorithms. However, the library lacks the test cases needed for the article. Therefore, this section compares the performance of PSO, standard QPSO, and hybrid QPSO by adapting the resource-constrained multi-mode project scheduling test cases in PSPLIB and using their solution results as the basis.

### Comparison experiments of multiple algorithms running independently based on different case sizes

(1)Algorithm parameter setting.

To verify the advantages of this algorithm in terms of result quality and convergence speed, a mainstream commercial computer (Windows 10 operating system, 8 GB RAM, Intel(R) Core (TM) i5-8300H CPU @ 2.30 GHz, with RM8.00 GB) was used to program the hybrid quantum particle swarm algorithm and the standard quantum particle swarm algorithm in MATLAB R2020a as described in this paper. The algorithm parameter settings are as follows: in the standard particle swarm algorithm, the population size is 100, the inertia weight is [0.8 0.4], cc = [1.5,1.5], and the particle velocity range is [− 0.5, 0.5]. In the hybrid quantum particle swarm algorithm, as well as the standard quantum particle swarm algorithm, the population size is 100, the number of iterations of all three algorithms is 200, the number of independently run experiments is 450, and the optimal solution is retained for each iteration.

## (2) Test Results and Discussion

**Figure 6:**
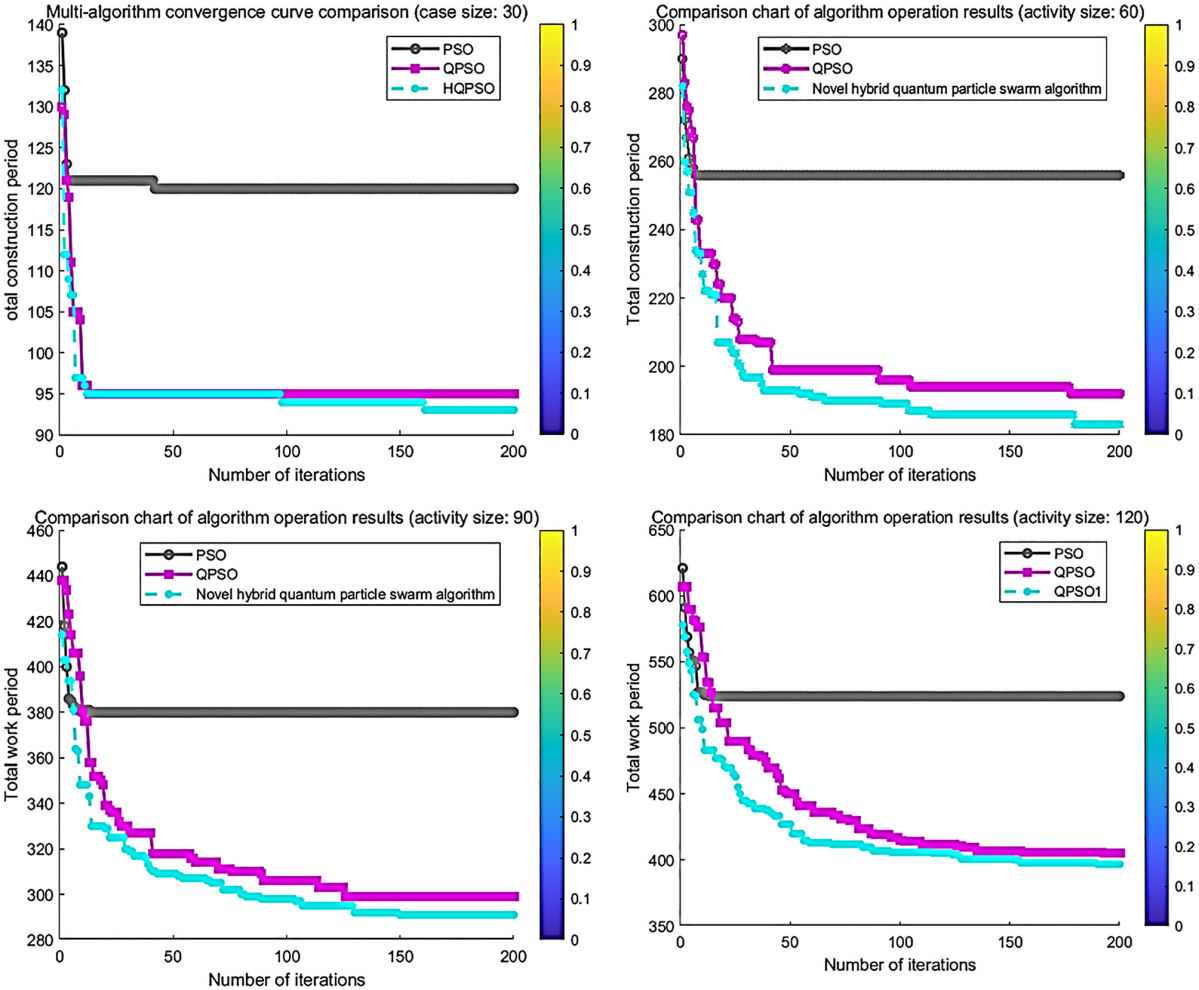
Convergence curve comparison chart.

Table 2 and Fig. 6 present the experimental results of multiple independent runs of various algorithms based on different case sizes and the number of resources. The data in the table demonstrates that, at different case sizes, the traditional QPSO outperforms PSO in terms of global search performance. However, it still suffers from premature aging. On the other hand, the hybrid QPSO algorithm, which incorporates JAYA optimized search, achieves better solution quality compared to the other two algorithms. Furthermore, the convergence curves in the figure reveal that the hybrid algorithm also outperforms the other two algorithms in terms of convergence performance. Nonetheless, for the same case size, the values obtained by each algorithm increase as the number of resources decreases, indicating a decrease in the number of skills and an increase in the resource skill constraint.

**Table 2:** Comparison experimental results of multiple algorithms running independently.

Case Size	Number of resources	Algorithm name	Optimal solution	worst solution	Average value	Standard deviation
30	25	PSO	90	112	103.3333	6.7259
		QPSO	82	93	89.4667	3.1366
		Hybrid QPSO	83	88	87.533	1.6421
	20	PSO	95	115	105.4000	5.8039
		QPSO	85	96	88.9333	3.8816
		Hybrid QPSO	84	90	87.5333	1.8752
60	25	PSO	219	253	236.7333	9.1688
		QPSO	187	193	189	2.1044
		Hybrid QPSO	183	186	184.6667	1.2909
	20	PSO	222	256	233.4000	9.3049
		QPSO	188	194	190.8667	4.6116
		Hybrid QPSO	184	187	185.9333	1.3045
90	25	PSO	337	393	374.2	14.4479
		QPSO	290	304	296.0667	2.7506
		Hybrid QPSO	288	293	290.5333	1.5522
	20	PSO	367	407	380.0667	19.8667
		QPSO	293	325	300.1333	3.2530
		Hybrid QPSO	289	306	292.2000	1.7329
120	25	PSO	492	580	533.6	20.7082
		QPSO	397	410	403.3333	3.1519
		Hybrid QPSO	394	397	396.2	1.0823
	20	PSO	501	583	514.9333	22.9829
		QPSO	417	438	417.0667	5.7795
		Hybrid QPSO	397	413	406.4000	2.7599

## Example applications

### Project Introduction

**Figure 7:**
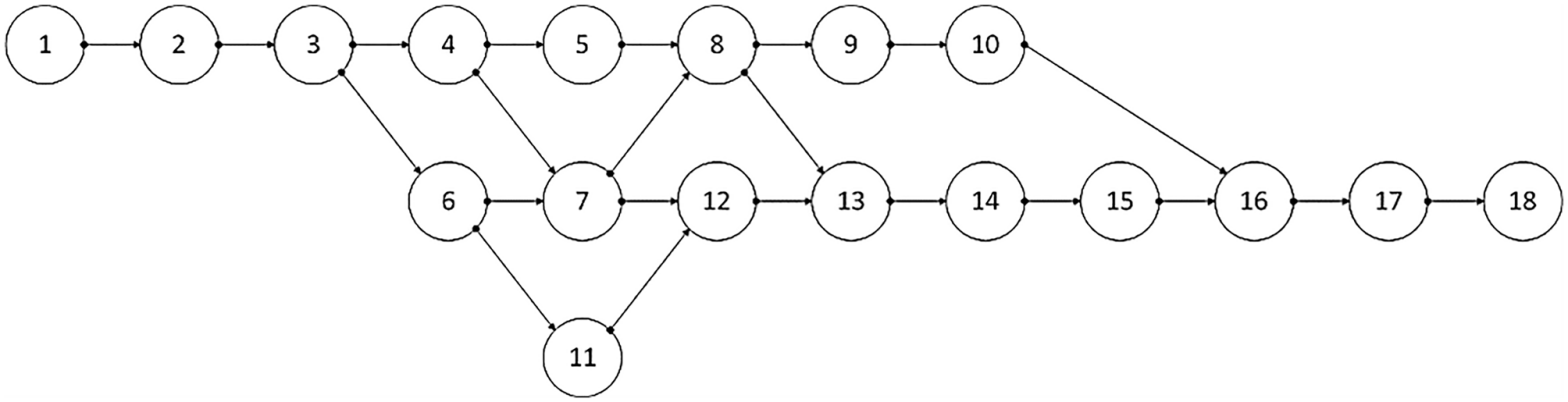
Results of hybrid quantum particle swarm algorithm runs.

The project is a continuous rigid bridge girder project in Changsha, Hunan Province, with a duration of 63 days. The project process information is shown in Table 3. Figure 7 represents the project logic network diagram.

**Table 3:** Project process information.

Work Number	Process name	Model number	Duration	Pre-tightening process	Skill 1	Skill 2	Skill 3	Skill 4
1	Virtual Process	1	0		0	0	0	0
2	Earth excavation	1	2	1	0	5	0	3
		2	4		6	0	0	3
		3	5		3	0	0	3
3	No.0 bridge deck reinforcement tying	1	3	2	7	0	3	0
		2	5		5	0	0	8
		3	6		4	0	1	0
4	No. 0 bridge deck formwork installation	1	4	3	0	5	0	8
		2	5		0	5	0	5
		3	7		0	4	3	0
5	No.0 bridge abutment concrete pouring	1	1	4	0	4	4	0
		2	2		6	0	4	0
		3	2		4	0	0	5
6	No.1 pier column bearing platform and pier reinforcement tying	1	6	3	0	8	8	0
		2	7		0	8	0	6
		3	8		0	6	6	0
7	Pier 1 bearing platform and pier formwork installation	1	3	4, 6	9	0	4	0
		2	4		0	10	0	7
		3	6		7	0	0	5
8	Pouring of concrete for pier column bearing and pier body of No.1 pier	1	1	5, 7	0	9	8	0
		2	2		4	0	8	0
		3	2		0	6	0	3
9	Scaffolding erection of continuous beam between No.0 abutment and No.1 pier column	1	4	8	0	9	0	6
		2	6		0	8	0	4
		3	7		3	0	7	0
10	Formwork installation of continuous beam of No.0 abutment and No.1 pier column	1	3	9	0	8	6	0
		2	4		4	0	2	0
		3	6		4	0	0	5
11	No.2 bridge abutment reinforcement tying	1	5	6	0	4	10	0
		2	6		6	0	8	0
		3	8		0	2	0	4
12	No.2 bridge deck formwork installation	1	4	7, 11	1	0	8	0
		2	5		0	3	6	0
		3	7		0	2	3	0
13	No.2 abutment concrete pouring	1	1	8、12	1	0	2	0
		2	1		1	0	0	4
		3	1		0	5	2	0
14	Scaffolding erection of continuous beam between No.1 abutment and No.2 pier column	1	5	13	0	7	0	3
		2	6		0	5	0	1
		3	8		0	3	6	0
15	Formwork installation of continuous beam of No.1 abutment and No.2 pier column	1	4	14	5	0	10	0
		2	5		0	3	0	1
		3	7		4	0	9	0
16	Continuous beam reinforcement tying	1	5	10、15	0	5	0	7
		2	7		2	0	5	0
		3	9		0	4	5	0
17	Continuous beam concrete pouring	1	1	16	4	0	3	0
		2	2		0	7	3	0
		3	2		0	6	0	3
18	Virtual Process	1	0	17	0	0	0	0

### Algorithm operation results and analysis

(1)Results of hybrid quantum particle swarm algorithm runs.

**Figure 8:**
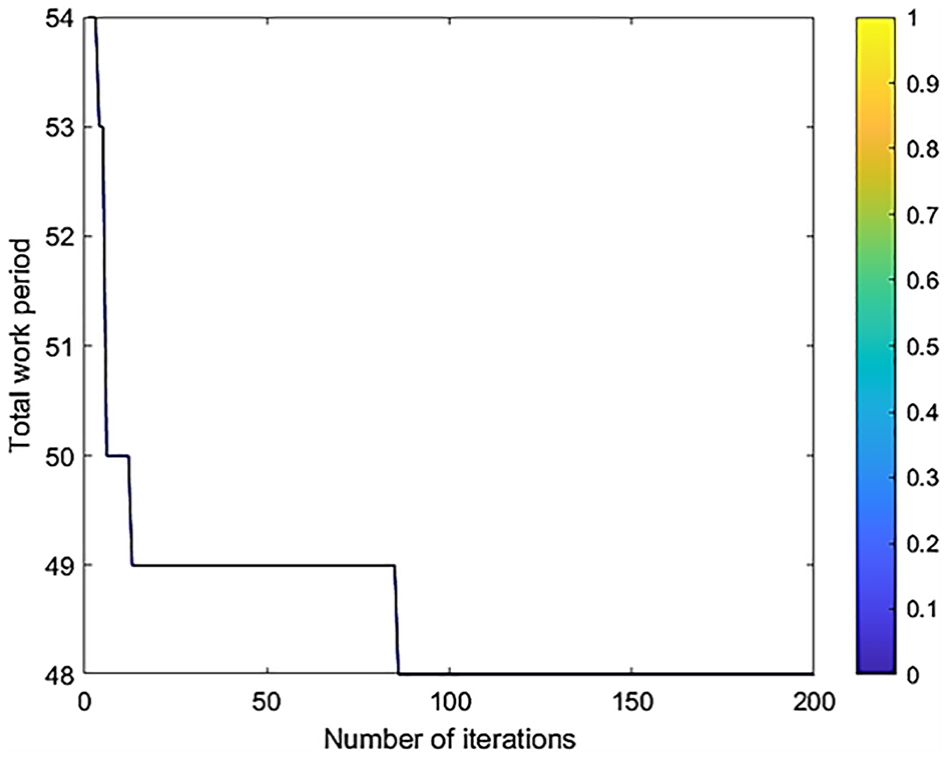
Results of hybrid quantum particle swarm algorithm runs.

With the consideration of multiple execution modes of project activities and multiple skill resources, the hybrid quantum particle swarm algorithm can be used to obtain the minimum project duration of 48 days, which is 15 days less than the original time limit, thus verifying the effectiveness of the algorithm. The results of the algorithm run are shown in Fig. 8 (the algorithm parameters are the same as in the performance experiments).

(2)Scheduling optimization results and analysis.

Based on the process information, Table 4 further gives the process mode selection, different resources, and capabilities allocation obtained by the hybrid quantum particle swarm algorithm. For example, process 10 takes mode 2 and selects the 2nd capability of resource 2 for execution. As described in the literature review, the scheduling method proposed in this paper not only considers resource constraints compared to traditional scheduling techniques but also uses a hybrid quantum particle swarm algorithm to solve the scheduling problem. At construction sites, the table can be used as a reference for managers to schedule construction personnel in advance, thus effectively avoiding human resource conflicts.

**Table 4:** Project scheduling strategy table.

Process	Mode	Start time	Ending time	Multi-capability resources number (Skill number)
1	1	0	0	Virtual process
2	1	0	2	1(2).4(2).17(2).18(2).21(2).22(4).23(4).25(4)
3	1	2	5	1(1).2(1).4(1).7(1).8(1).11(1).14(1).15(3).18(3).20(3)
4	1	16	20	1(2).2(2).4(2).6(2).9(2).11(4).13(4). 16(4).17(4).21(4).22(4).23(4).25(4)
5	1	20	21	2(2).3(2).4(2).8(3).11(2).13(3).18(3).21(2).23(3)
6	1	5	11	1(2).2(2).3(1).4(2).7(2).8(2).10(2).11(2).13(3).15(3).17(3).20(3).21(3).23(3).24(3).25(3)
7	1	21	24	1(1).2(1).3(2).7(1).10(1).11(1).12(1).13(1).14(1).15(3).18(3).20(3).24(3)
8	1	28	29	1(2).2(2).3(2).5(2).7(2).8(2).9(2).10(2).11(2).12(3).13(3).15(3).20(3).23(3).24(3).25(3)
9	1	29	33	2(2).4(2).5(2).6(2).9(2).11(2) .12(2).13(2).14(2).16(4).20(4).21(4) .22(4).23(4).25(4)
10	2	33	37	1(1).2(1).3(1).7(1).10(3).15(3)
11	1	11	16	1(2).2(2).3(2).7(3).8(3).10(3).11(3).13(3).15(3).20(3).23(3).24(3)
12	1	24	28	2(1).3(3).7(3).8(3).10(3).13(3).15(3).20(3).23(3)
13	1	29	30	3(1).10(3).15(3)
14	1	33	38	4(2).5(2).6(2).11(2).16(2).17(2).20(2).21(4).23(4).25(4)
15	1	38	42	1(1).2(1).3(1).4(1).7(1).10(3).11(3).13(3).14(3).15(3).18(3).20(3).23(3).24(3)
16	1	42	47	1(2).4(2).5(2).6(2).7(1).11(4).14(4).16(4).17(4).18(4).21(4).25(4)
17	1	47	48	1(1).3(1).11(1).13(1).14(3).15(3).20(3)
18	1	48	48	Virtual Process

(3)Comparison of the results of multiple algorithms runs.

**Figure 9:**
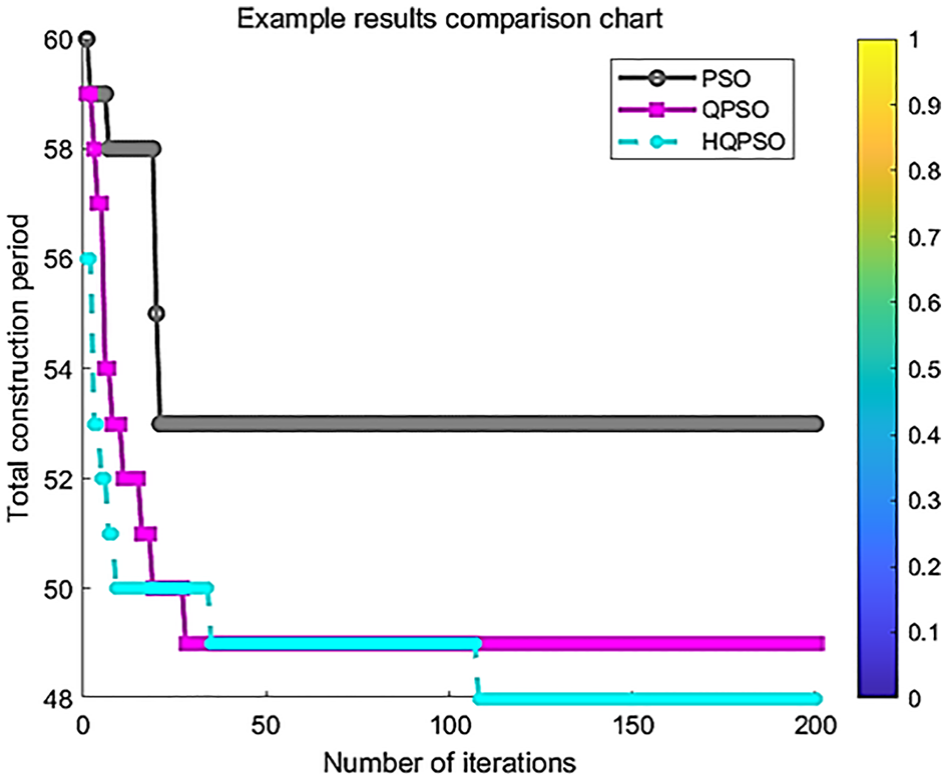
Multi-algorithm convergence curve comparison chart.

The Fig. 9 shows the comparison of convergence curves of various algorithms under practical cases. From the figure, it can be seen that the convergence curve of the hybrid quantum particle swarm has a more obvious downward trend in practical applications and is closer to the optimal solution of the optimization objective than the traditional QPSO and PSO algorithms.

### Management opinion

In addition to practical exploration, the article, based on the problems and findings of scheduling multi-modal projects with multi-skilled resource constraints, can make the following observations to facilitate effective resource management by managers:

Optimize skill matching: Ensure appropriate alignment between required tasks and the skills possessed by team members. By thoroughly understanding project requirements and the skill levels of team members, tasks can be allocated more effectively, leading to improved work efficiency.

Strengthen resource management: Effectively manage and utilize multi-skilled resources, including sharing resources across departments or teams. Establish a resource repository to record the skills and availability of each member, enabling accurate scheduling when needed.

Harness intelligent technologies: Explore and apply intelligent technologies and algorithms such as machine learning and optimization algorithms to assist in project scheduling decisions. These technologies can provide more accurate predictions, optimize resource allocation, and offer real-time decision support during the scheduling process.

Continuous improvement and learning: Regularly evaluate the project scheduling process and learn from experiences. Collaborate with team members, collect feedback, and continuously improve scheduling strategies and methods to enhance the effectiveness and efficiency of scheduling.

In summary, by optimizing skill matching, enhancing resource management, and leveraging intelligent technology, managers can effectively address the challenges associated with scheduling multi-modal projects under multi-skilled resource constraints.

## Conclusion

The article proposes an optimization model as well as a hybrid quantum algorithm for the multi-skill resource-constrained multimodal project scheduling-based problem. Based on the process logic relationship and the list of process capability requirements, the proposed hybrid quantum algorithm (HQPSO) can arrange resources and capabilities in activity or time order to meet the resource constraints and minimize the project duration during the scheduling process. Additionally, two experiments are designed to verify the superiority and effectiveness of the improved hybrid quantum algorithm: Experiment 1, in which multiple algorithms are run independently based on different test case sizes to verify the superiority and stability of the proposed algorithm; Experiment 2, in which the improved algorithm is applied to solve the problem based on real cases to verify its effectiveness. The experimental results show that the hybrid algorithm has superior solution performance and can provide targeted resource scheduling strategies based on multi-skilled resource-constrained multi-modal project scheduling problems during project process execution, which can help contractors to achieve near-optimal project goals. The algorithm developed in this study has a better ability to solve the project scheduling problem compared to conventional algorithms. In addition, in response to the results of the experiment, the article provides practical insights derived from real-world scenarios. These insights can serve as a valuable guide for managers to effectively manage resources. The results reported in the article can be used as a benchmark for the remaining researchers' experiments.

Limitations and suggestions for future research: Since the article does not address the impact of environmental uncertainty on project scheduling, future research endeavors will take into account various sources of uncertainty, including the social and political environment. Through a comprehensive factor analysis, the aim is to gain a deeper understanding of how these uncertainties affect resource-constrained project scheduling, ultimately contributing to the improvement of project scheduling theory.

## Data Availability

The datasets used and/or analysed during the current study available from the corresponding author on reasonable request.
